# Targeting Ubiquitin-Specific Protease 7 (USP7) in Cancer: A New Insight to Overcome Drug Resistance

**DOI:** 10.3389/fphar.2021.648491

**Published:** 2021-04-22

**Authors:** Jiabin Lu, He Zhao, Caini Yu, Yuanyuan Kang, Xiaochun Yang

**Affiliations:** Center for Drug Safety Evaluation and Research, College of Pharmaceutical Sciences, Zhejiang University, Hangzhou, China

**Keywords:** tumor therapy resistance, ubiquitination modification, ubiquitin-proteasome system, ubiquitin-specific protease 7, deubiquitinases, USP7 inhibitors

## Abstract

Chemoresistance is one of the leading causes for the failure of tumor treatment. Hence, it is necessary to study further and understand the potential mechanisms of tumor resistance to design and develop novel anti-tumor drugs. Post-translational modifications are critical for proteins’ function under physiological and pathological conditions, among which ubiquitination is the most common one. The protein degradation process mediated by the ubiquitin-proteasome system is the most well-known function of ubiquitination modification. However, ubiquitination also participates in the regulation of many other biological processes, such as protein trafficking and protein-protein interaction. A group of proteins named deubiquitinases can hydrolyze the isopeptide bond and disassemble the ubiquitin-protein conjugates, thus preventing substrate proteins form degradation or other outcomes. Ubiquitin-specific protease 7 (USP7) is one of the most extensively studied deubiquitinases. USP7 exhibits a high expression signature in various malignant tumors, and increased USP7 expression often indicates the poor tumor prognosis, suggesting that USP7 is a marker of tumor prognosis and a potential drug target for anti-tumor therapy. In this review, we first discussed the structure and function of USP7. Further, we summarized the underlying mechanisms by which tumor cells develop resistance to anti-tumor therapies, provided theoretical support for targeting USP7 to overcome drug resistance, and some inspiration for the design and development of USP7 inhibitors.

## Introduction

Cancer is a significant public health problem worldwide, which seriously threatens patients’ health and lives and brings a heavy burden to individuals, families and society (Chen et al., 2016; Siegel et al., 2021). With the emergence and development of traditional chemotherapy, radiotherapy, targeted therapy, immunotherapy and other medical technologies, some tumors have been effectively controlled and even cured. However, clinically there are still phenomena such as poor efficacy, tumor recurrence, metastasis, and drug resistance remain (Holohan et al., 2013; Quail and Joyce, 2013; Mahvi et al., 2018). Statistically, chemoresistance is one of the main causes of tumor treatment failure, which significantly limits the choice and use of anti-tumor drugs in the clinic and breaks cancer patients’ hope time and time again. Hence, it is necessary to study further and understand the potential mechanisms of tumor resistance to design and develop novel anti-tumor drugs.

Post-translational modification (PTM) of proteins is an important way to regulate protein structure, and is critical for proteins’ function under physiological and pathological conditions (Dai and Gu, 2010). Protein PTMs include phosphorylation, ubiquitination, acetylation, methylation, glycosylation, SUMOylation, and so on. Protein ubiquitination is one of the most common forms of PTM. Ubiquitin is a highly conserved endogenous protein consisting of 76 amino acids and widely exists in eukaryotic cells, from where it got the name “ubiquitin”. Ubiquitination refers to a reversible modification process in which ubiquitin molecules specifically covalently bind to target protein residues identified from cellular protein molecules under the catalysis of a series of special enzymes (Mansour, 2018).

The ubiquitin-proteasome system (UPS) is an intracellular non-lysosomal protein degradation pathway. Under the catalysis of the ubiquitin-activating enzymes (E1s), the ubiquitin-conjugating enzymes (E2s) and the ubiquitin ligases (E3s), the ubiquitin covalently binds to the targeted protein, leading to its degradation by the 26S proteasome complex. Due to the large variety of substrate proteins modified by ubiquitination, therefore, the UPS pathway plays a critical part in diverse cellular processes, including cell proliferation, apoptosis, differentiation, gene expression, transcription regulation, signal transduction, damage repair, inflammation, and immunity (Narayanan et al., 2020). The protein degradation process mediated by UPS is the most well-known function of ubiquitination modification. However, ubiquitination also participates in the regulation of many other biological processes, such as protein trafficking, protein-protein interaction, kinase activity regulation, receptor response, DNA replication and repair, gene transcription, etc. (Leslie et al., 2016; Mansour, 2018).

Correspondingly, a group of proteins named deubiquitinases (DUBs) can hydrolyze the isopeptide bond and disassemble the ubiquitin-protein conjugates, thus preventing substrate proteins from degradation or other biological processes. Ubiquitin-specific protease 7 (USP7), also known as herpesvirus-associated ubiquitin-specific protease (HAUSP), is one of the most extensively studied DUBs. A large number of previous studies have found that USP7 exhibits a high expression signature in a variety of malignant tumors, including myeloma (Chauhan et al., 2012), prostate (Qi et al., 2020), hepatocellular (Cai et al., 2015), ovarian cancer (Ma and Yu, 2016) and glioma (Cheng et al., 2013). Also, the increased USP7 expression often indicates a poor tumor prognosis. As a result, some scholars considered USP to be a marker of tumor prognosis and a potential drug target for anti-tumor therapy (Wang et al., 2019).

Furthermore, we have also observed that USP7 is closely related to the emergence of anti-tumor drug resistance. Therefore, in this review, we will start from the structure and function of USP7, reveal the inextricable connection between USP7 and tumor resistance, and propose that targeting USP7 might be a new insight to overcome drug resistance.

## The Structure and Function of the Human Ubiquitin-Specific Protease 7

**FIGURE 1:**
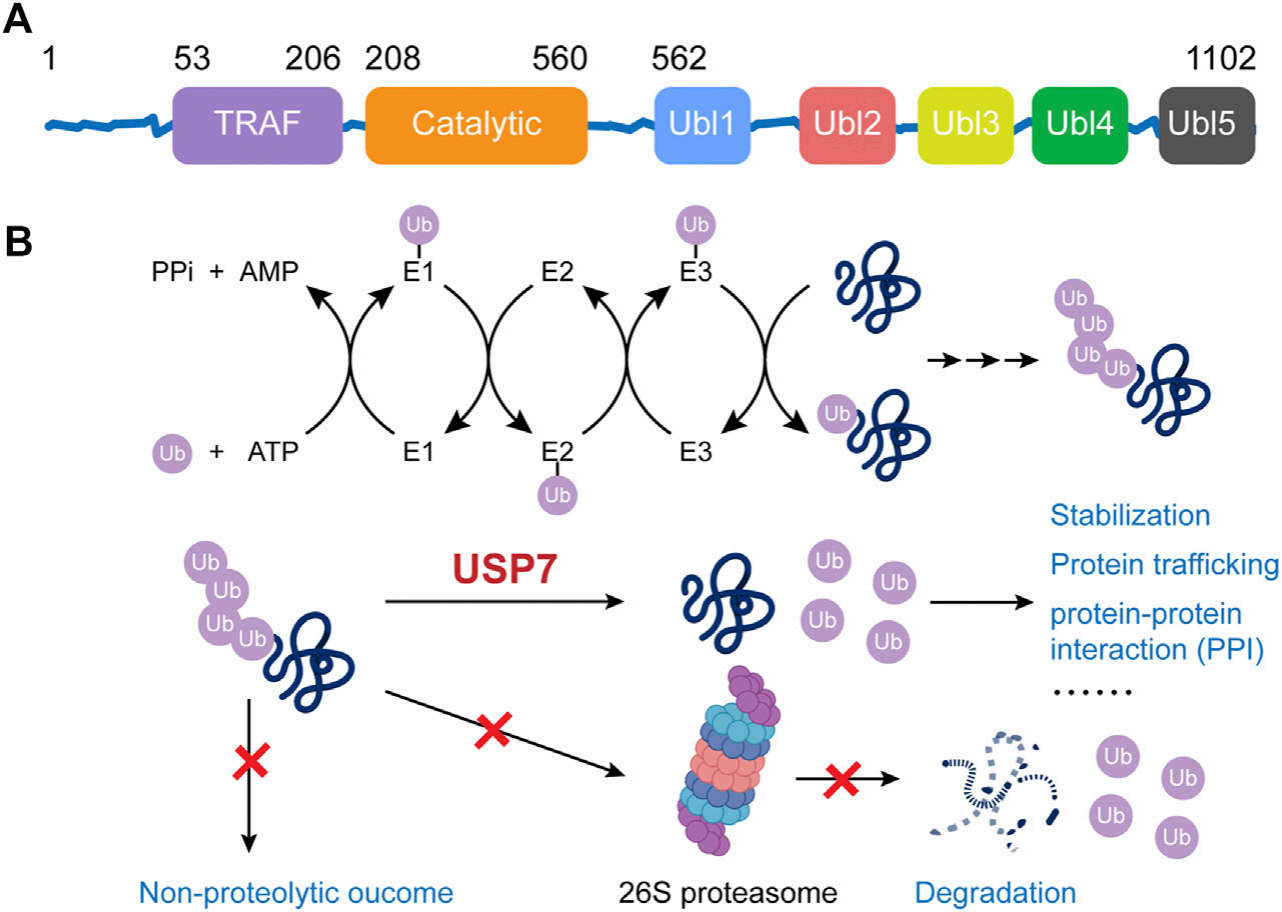
The structure and function of USP7.

The human *USP7* gene is located on chromosome 16p13.2, and it encodes the USP7 protein, which consists of 1,102 amino acids. USP7 protein contains three major domains, including a N-terminal tumor necrosis factor receptor-associated factor (TRAF) domain (amino acids 53–206), a central catalytic domain (amino acids 208–560), and a C-terminal tandem ubiquitin-like (Ubl) domain (amino acids 560–1,102) (Figure 1A) (Hu et al., 2006). Among them, the TRAF domain can directly bind with the substrates via the P/AxxS motifs and it can also regulate the nuclear localization process of USP7 to a certain extent. The catalytic core of USP7 includes three conserved functional areas (Fingers, Palm, and Thumb), where an anti-parallel β-sheet structure creates a deep cleft for ubiquitin-binding. The catalytically active Cys box (∼19 amino acids) and His box (60–90 amino acids) locate at the opposing sides of the cleft, between the Palm and Thumb domain (Hu et al., 2002). The C-terminal Ubl domain contains five Ubl-subunits arranging in a 2–1–2 manner (Ubl1/2, Ubl3, and Ubl4/5), which ultimately enhances the binding ability of USP7 for substrates and its deubiquitinating activity. Specifically, Ubl1/2/3 contribute to the allosteric transition partly by activating GMP synthetase while Ubl4/5 can interact with the switching loop in the catalytic domain and help rearrange the catalytic triad to an active conformation (Faesen et al., 2011).

The role of USP7 is to deubiquitinate the ubiquitinated substrate proteins, thereby preventing them from ubiquitination-dependent proteasome degradation or ubiquitination-mediated protein trafficking, consequently stabilizing them or maintaining their subcellular localization (Figure 1B). The Fingers precisely coordinate the ubiquitin N-terminal residues at the catalytic domain of USP7 and guided the C terminus into the catalytic cleft between the Palm and the Thumb, which is rich in acidic amino acids (Hu et al., 2002; Molland et al., 2014; Turnbull et al., 2017). There’s a unique structural feature, known as the switching loop, located between helices α4 and α5 of USP7, by which the catalytic core undergoes conformational changes, rearranging the catalytic triplet (Cys223, His464, and Asp481) to close proximity, thus allowing ubiquitin interaction (Hu et al., 2002; Molland et al., 2014). Upon the binding, the Phe409 side chain in the binding channel rotates to open a hydrophobic pocket to accommodate ubiquitin (Turnbull et al., 2017). The isopeptide bonds between ubiquitin and substrates would be hydrolyzed through a three-step mechanism: binding, acylation, and deacylation. Initially, USP7 binds with the substrate proteins, undergoing the mentioned conformational changes. Afterward, the highly conserved catalytic Cys223 is deprotonated by a histidine residue, resulting in a nucleophilic attack by deprotonated sulfhydryl of cysteine on the carbonyl carbon atom of glycine 76 on ubiquitin to generate an acyl-enzyme intermediate. Finally, the intermediate is hydrolyzed to release free enzymes and ubiquitin (Daviet and Colland, 2008). A recent study reported that USP7 preferentially interacts with and cleaves ubiquitin moieties with free Lys48 side chains rather than Lys63 ones (Kategaya et al., 2017).

An accurate characterization of the structure of USP7 and a deep understanding of its function could help better explain the relationship between USP7 and tumorigenesis, progression, and the phenomenon of drug resistance; also, these characterizations may bring new insights for developing new-type USP7-targeted drugs with higher potency.

## Ubiquitin-Specific Protease 7 is Closely Associated With Anti-Tumor Therapies Resistance

Now, based on a large number of scientific observations and studies, we can make clear that USP7 plays a vital role in the development of drug resistance in multiple tumors in response to the threat of therapeutic agents. While the specific signaling pathways regulated by USP7 may differ in the above processes, the consequence is always that the deubiquitinating activity of USP7 leads to an aberrant fate of substrate proteins and consequent involvement in the generation of drug resistance. This section will enumerate examples of USP7’s involvement in anti-tumor therapy resistance to demonstrate USP7 as a biomarker for the phenomenon of tumor resistance and an important prognostic indicator for tumor therapy.

### Proteasome Inhibitors

Multiple myeloma (MM) is one of the most frequent hematological malignancies characterized primarily by the abnormal synthesis and secretion of monoclonal immunoglobulins. In recent years, proteasome inhibitors, which could induce tumor cell death by inhibiting the proteasome’s function and leading to the accumulation of abnormal proteins in cells, were approved by the FDA to treat MM and have significantly improved the clinical outcome of MM patients. Among them, bortezomib (BTZ; brand name: Velcade) was the first-in-class proteasome inhibitor for the treatment of MM, and it can dramatically induce MM cell death by stabilizing IκBα protein to inhibit the activation of NF-κB signaling pathway (Yao et al., 2018). However, MM is a complex disease, and the drug-resistant subclones that appear in many patients during therapy can still lead to treatment failure or tumor relapse (Xia et al., 2020). Therefore, it is necessary to investigate the underlying mechanisms by which MM develops resistance to proteasome inhibitors and further target those resistance mechanisms for selective intervention or remediation.

As early as 2012, an interesting study showed that the HDM2/p53/p21 signaling axis is involved in MM cells survival during BTZ therapy. HDM2 is a primary substrate of USP7, which can bind to the tumor suppressor protein p53 to exert its E3 enzymatic activity and drive p53 ubiquitination and subsequent degradation. In general, the higher the HDM2 expression, the worse the tumor prognosis. Since mutations or deletions of p53 confer MM cells survival, activation or stabilization of p53 may offer a novel therapeutic strategy. As expected, both genetic ablation of USP7 gene (siRNA or somatic knockout) and pharmacological inhibition of USP7 protein (P5091) prevents USP7 from deubiquitinating HDM2, resulting in stabilization and accumulation of p53, as well as p21-induced growth arrest and cell death (Chauhan et al., 2012). Another study had found that high expression of USP7 in MM is a prognostic marker of short overall survival and poor outcome. USP7 knockout restored MM sensitivity to BTZ and induced apoptosis by stabilizing IκBα and blocking the NF-κB signaling pathway. Similarly, the treatment of USP7 inhibitors (P5091 or P22077) also suppressed the activation of NF-κB, and a combination of USP7 inhibitors and BTZ triggered the synergistic anti-tumor activity in BTZ-resistant MM cells (Yao et al., 2018).

Additionally, NEK2 was reported to be associated with bortezomib resistance in MM, and high expression of NEK2 confers inferior prognosis and survival in MM patients. Franqui-Machin et al. showed that USP7 could prevent NEK2 from undergoing ubiquitination-dependent degradation and stabilize it. The increased levels of NEK2 kinase activates the canonical NF-κB pathway via the PP1α/AKT axis, which facilitates p65 nuclear translocation to activate transcription of downstream target genes. Also, NEK2 can activate the secretory heparanase to destroy bone tissue in an NF-κB-dependent manner. Intriguingly, the USP7 inhibitor (P5091) significantly inhibited myeloma cells’ cell growth and overcame NEK2-induced and -acquired BTZ resistance (Franqui-Machin et al., 2018). A recent study showed that NEK2 enhances autophagy and induces BTZ resistance in MM cells through stabilization and up-regulation of Beclin-1. Mechanistically, NEK2 binds to both Beclin-1 and USP7 in MM cells, and NEK2 stabilizes Beclin-1 through USP7-mediated deubiquitination. However, the underlying mechanisms by which NEK2, Beclin-1, and USP7 regulate each other remain unclear, which need to be further studied and revealed (Xia et al., 2020).

### Poly-(ADP)-Ribose Polymerase Inhibitors

PARP inhibitors work based on the principle of synthetic lethality, to which homologous recombination (HR)-deficient tumors harboring mutations in BRCA1/2 or other HR factors are hypersensitive (Noordermeer and van Attikum, 2019). In detail, PARP inhibitors can inhibit the repair process of DNA single-strand damage, which can be converted into double-strand break (DSB) during the formation of DNA replication fork, and HR is a pathway essential for DSBs repair. For those tumors with defects in HR repair, DSBs cannot be repaired, eventually resulting in cell death. Unfortunately, intrinsic and acquired resistance to PARP inhibitors has become a significant problem in the clinic (Noordermeer and van Attikum, 2019).

CCDC6 is an oncoprotein, of which deficiency affects DNA damage and repair processes and sensitizes tumor cells to the treatment of PARP inhibitors (like olaparib). Meanwhile, CCDC6 was reported to be one of the substrates of USP7, which can prevent CCDC6 from ubiquitination and stabilize it. As expected, USP7 inhibitors (like P5091) can downregulate CCDC6 protein levels and favor tumor cells sensitivity to PARP inhibitors in non-small cell lung cancer (Morra et al., 2015), lung neuroendocrine cancer (Malapelle et al., 2017) and hormone-sensitive and castration-resistant prostate cancer (Morra et al., 2017).

### HER2 Inhibitors

Trastuzumab is a humanized monoclonal antibody that targets the extracellular binding site of the HER2 receptor and inhibits its downstream PI3K/Akt/mTOR and Ras/MAPK axis. PI3K/Akt pathway is one of the most critical carcinogenic pathways that block apoptosis and promote cell proliferation through upregulation of growth factor receptors (EGFR, IGF1R, HER2, etc.) or PTEN inactivation. PTEN is well-known to be one of the substrates of USP7. The deubiquitinating activity of USP7 is responsible for PTEN nuclear exclusion. It impairs its tumor-suppressive functions, resulting in the constant activation of PI3K/Akt signaling and drug resistance to HER2 inhibitor (trastuzumab) in HER2-positive breast carcinomas (Gallardo et al., 2012). It was also reported that USP7 inhibition (P5091) restores PTEN nuclear pool and its onto suppressive activity in chronic lymphocytic leukemia (Carrà et al., 2017).

### Other Targeted Therapies

USP7 was also associated with drug resistance to other targeted treatments in malignancies. For instance, several tyrosine kinase inhibitors were demonstrated to be more effective after inhibition of USP7 or MDM2 (Wang et al., 2017). USP7 inhibitor GNE-6640 was proven to enhance PIM2 ubiquitination and increase PIM kinase inhibitors’ cytotoxicity (Kategaya et al., 2017). Moreover, USP7 can up-regulate β-catenin, suggesting the potential of USP7 as a therapeutic target in colorectal cancer with a hyperactivated Wnt signaling, to suppress growth and overcome chemoresistance to Wnt inhibitors (An et al., 2017).

### Chemotherapy Drugs

#### Cell Cycle Specific Agents

Taxanes are a class of cytotoxic drugs that specifically act on the G2 and M phases of the cell cycle, mainly including paclitaxel, docetaxel, and cabazitaxel. Taxanes can promote the irreversible accumulation of tubulin and hinder the normal dynamic regeneration of microtubule bundles, resulting in abnormal attachment of kinetochores and chromosome partitioning during the formation of the mitotic spindle, thereby inhibiting cell division and proliferation in cancer cells (Giovinazzi et al., 2013). This phenomenon is also known as the mitotic catastrophe (Shin et al., 2020). Since the mitotic activity of tumor cells is far more active than that of normal cells, low-dose taxanes can effectively kill tumor cells without causing serious damage to normal tissues. Taxanes are the first-line chemotherapeutic agents in the clinic for several carcinomas of the breast, lung, prostate, and ovary. However, chemoresistance has become one major cause of poor efficacy and even death in clinical patients (Shin et al., 2020).

It is reported that USP7 can mediate the deubiquitination of CHFR protein, and the E3 enzyme activity of CHFR protein can target Aurora-A kinase for ubiquitination-dependent protein degradation. As a result, USP7 can mediate the chemoresistance of taxanes by regulating mitosis progression, and the inhibition of USP7 helps to enhance the taxane sensitivity of tumor cells (Giovinazzi et al., 2013). PLK1 was reported to be a novel substrate of USP7, and USP7 maintains the protein stability of PLK1. USP7 inhibition overcomes taxane resistance by inducing the protein degradation of PLK1, resulting in chromosome misalignment in mitosis (Peng et al., 2019). Also, Shin et al. demonstrated that a combination of inhibitors of USP7 (P22077) and the mitotic kinase PLK1 (volasertib) increased the sensitivity of paclitaxel-resistant lung cancer through down-regulation of MDR1/ABCB1 (Shin et al., 2020). Moreover, heterogeneous nuclear ribonucleoprotein A1 (hnRNPA1) was found to be a substrate of USP7, and USP7 stabilizes hnRNPA1 through the deubiquitinating activity. Paclitaxel and cisplatin promote miR-522 secretion from cancer-associated fibroblasts by activating USP7/hnRNPA1 axis, leading to arachidonate lipoxygenase 15 suppression and decreased lipid-ROS accumulation in cancer cells and ultimately promote acquired chemoresistance in gastric cancer (Zhang et al., 2020a).

Cytarabine is a pyrimidine anti-metabolite that acts explicitly on the S phase of the cell cycle. The cytarabine’s chemical structure is similar to the essential substance for DNA metabolism, which can specifically interfere with the normal synthesis of DNA, and inhibit cell division and proliferation. Cartel et al. found that CHK1 is a substrate of USP7, and USP7 modulates the protein stability of CHK1. Patients with a high USP7 expression were more prone to chemoresistance, and USP7 inhibition (P22077) acts in synergy with cytarabine to kill acute myeloid leukemia cell lines with high USP7 levels, indicating that USP7 is both a marker of chemoresistance and a potential therapeutic target in enhancing chemosensitivity (Cartel et al., 2021).

In addition, USP7 has been reported to be involved in chemoresistance to DNA damaging agents such as camptothecin (topoisomerase I inhibitor) and etoposide (topoisomerase II inhibitor) (Becker et al., 2008; Fan et al., 2013).

#### Cell Cycle Nonspecific Agents

Doxorubicin (DOX) is a cell cycle nonspecific agent that can directly act on DNA or intercalate DNA to interfere with DNA transcription, thereby preventing mRNA synthesis and causing cell death. Some studies have shown that USP7 is a key effector protein in the emergence of DOX chemoresistance in neuroblastoma, hepatocellular carcinoma and pancreatic cancer, and USP7 inhibition can significantly increase the chemosensitivity of tumors (Fan et al., 2013; Zhang et al., 2020a; Chen et al., 2020).

Moreover, USP7 has been reported to be involved in chemoresistance to x-ray irradiation or DNA damaging agents, including methyl methanesulfonate, cyclophosphamide, mitomycin, and neocarzinostatin (Zhang et al., 2014; Zhao et al., 2015; Wang et al., 2016; Agathanggelou et al., 2017; Su et al., 2018).

## Substrates of Ubiquitin-Specific Protease 7 Involved in Anti-Tumor Therapies Resistance

A wide range of proteins have been identified as the potential substrates and binding partners of USP7, including p53, PTEN, CHK1, CHFR, and so on, most of which and their downstream signaling cascades are necessary for DNA repair, epigenetic control, tumor suppression and immune response (Wang et al., 2019). Overexpression of USP7 impairs the ubiquitination-dependent proteasome degradation or protein trafficking of these proteins, thus impairing drug efficacy and even leading to drug resistance. This section further summarized and arranged the substrate proteins regulated by USP7 involved anti-tumor therapies resistance (Table 1).

**TABLE 1:** Substrates of USP7 involved in anti-tumor therapies resistance.

Substrates of USP7	Therapies resistance	Tumors	References
ALKBH3	Alkylating agents	−	Zhao et al. (2015)
Beclin-1	Bortezomib	Multiple myeloma	Xia et al. (2020)
CCDC6	PARP-inhibitors	Lung neuroendocrine cancer	Malapelle et al. (2017)
CCDC6	PARP-inhibitors	Hormone-sensitive and castration-resistant prostate cancer	Morra et al. (2017)
CCDC6	−	Non-small cell lung cancer	Morra et al. (2015)
CHFR	Taxanes	−	Giovinazzi et al. (2013)
CHK1	Cytarabine	Acute myeloid leukemia	Cartel et al. (2021)
CHK1	Chemotherapy and radiotherapy	Breast cancer	Zhang et al. (2014)
HDM2	Bortezomib	Multiple myeloma	Chauhan et al. (2012)
HDM2	Camptothecin	Colon carcinoma	Becker et al. (2008)
HDM2	Doxorubicin and etoposide	Neuroblastoma	Fan et al. (2013)
hnRNPA1	Paclitaxel and cisplatin	Gastric cancer	Zhang et al. (2020a)
MDC1	DNA damage agents	Cervical cancer	Su et al. (2018)
NEK2	Bortezomib	Multiple myeloma	Franqui-Machin et al. (2018)
PHF8	Chemotherapy and radiotherapy	Breast cancer	Wang et al., (2016)
PIM2	PIM kinase inhibitors	−	Kategaya et al. (2017)
PLK1	Paclitaxel	Lung cancer	Shin et al. (2020)
PTEN	HER2 inhibitor (trastuzumab)	HER2-positive breast carcinomas	Gallardo et al. (2012)
RAD18	Chemotherapeutic agents	Chronic lymphocytic leukemia	Agathanggelou et al. (2017)
β-catenin	Wnt inhibitors	Colorectal cancer	An et al. (2017)
−	Bortezomib	Multiple myeloma	Yao et al. (2018)
−	Doxorubicin	Hepatocellular carcinoma	Zhang et al. (2020b)
−	Doxorubicin	Pancreatic cancer	Chen et al. (2020)

## Ubiquitin-Specific Protease 7 is a Potential Therapeutic Target in Overcoming Drug Resistance

Since USP7 has crucial roles in generating anti-tumor drug resistance, numerous studies have been conducted to pharmacologically inhibit its deubiquitinating activity from avoiding abnormal stabilization or trafficking of its substrates. Up to now, more than 160 small-molecule inhibitors of USP7 have been found.

The first lead-like inhibitor of USP7 (HBX41108) was reported in 2009, but its effect lacks specificity and may affect unrelated thiol proteases and additional deubiquitinating enzymes (Colland et al., 2009). As the conformation of the catalytic core domain of USP7 was accurately resolved and characterized (Hu et al., 2002), a generation of specific inhibitors, including HBX19818, HBX28258, P5091, P22077, and P50429, was developed and identified. These inhibitors mainly bind to the highly conserved cysteine residue Cys223 in the catalytic domain of USP7, which can trap the C-terminus of ubiquitin into the active pocket, thereby inhibiting the conformational transition and ultimately depriving USP7 of its deubiquitinating activity. Other types of USP7 inhibitor, including GNE-6640, GNE-6676, XL188, L55, FT671, and FT827, function in an allosteric manner. These allosteric inhibitors interact with adjacent regions rather than the catalytic triad, preventing the alignment of the catalytic triad and blocking ubiquitin-binding channels. The allosteric regulatory interaction site, also known as the switching loop, which allows sufficient space for compound binding, is on the Thumb domain of USP7.

### Covalent Catalytic Site Inhibitors

HBX19818 and HBX28258 covalently bind to catalytic Cys223 of USP7, of which basic amino group electrostatic interacting with Asp295 and Glu298 at the entrance of the ubiquitin-binding pocket (Reverdy et al., 2012). Another leading inhibitor, P5091, was identified in 2012 (Weinstock et al., 2012), and further optimization of it led to the discovery of P22077 and P50429. Both inhibitors selectively and covalently modify the catalytic C223 residue, perturbing BL1, BL2, and SL, which form the catalytic cleft and realign the active site upon enzyme activation (Chen et al., 2017; Pozhidaeva et al., 2017).

### Non-Covalent Allosteric Inhibitors

FT671 (Turnbull et al., 2017; Gavory et al., 2018) and XL188 (Lamberto et al., 2017; Schauer et al., 2020), with their heteroaromatic groups (PyrzPPip in FT671 and quinazolinone ketone in XL188, respectively) and piperidinol amide groups, form hydrogen-bonds within the S4-S5 pocket between the Palm and Thumb area of enzyme about 5.5Å from Cys223, and partially protrudes into the channel normally occupied by the C-terminal tail of ubiquitin, creating a steric clash. XL188 has been proven to protect the BL1 and α-4/5 loops surrounding the S4-S5 pocket from exchanging. The switching loop of USP7 enables unique positions of residues Tyr465 and Tyr514, which allows sufficient space for FT671 binding. FT671 based yield L55’s structural optimization shows a distinctive pose of binding, with a large upshift of Phe409 residue (Li et al., 2020). Aminopyridinephenolic non-covalent inhibitors GNE-6640 and GNE-6676 were identified in 2017 (Di Lello et al., 2017; Kategaya et al., 2017), whose target is the pocket at the Palm domain, approximately 12Å from the catalytic triad, preventing transition of the α5 helix to the active conformation. GNE-6640 owns higher and leads to apoptosis, while GNE-6676 mainly triggers cell cycle arrest.

### Covalent Allosteric Inhibitors

The first inhibitor, HBX41108, interacts with the enzyme-substrate complex at the pocket close to the ubiquitin-binding site that exists exclusively in the ubiquitin-bound conformation, and its chloro-substituent is interacting with a hydrophobic subsite (Colland et al., 2009). XL177A, an analog of XL188, exhibits higher efficacy and enhances exchange in the region from α2 to α4 of USP7 due to its covalent bond with Cys223 in addition to interactions same with its non-covalent analogue XL188 (Schauer et al., 2020), as is the case with FT827 and FT671 (Turnbull et al., 2017; Gavory et al., 2018).

**FIGURE 2:**
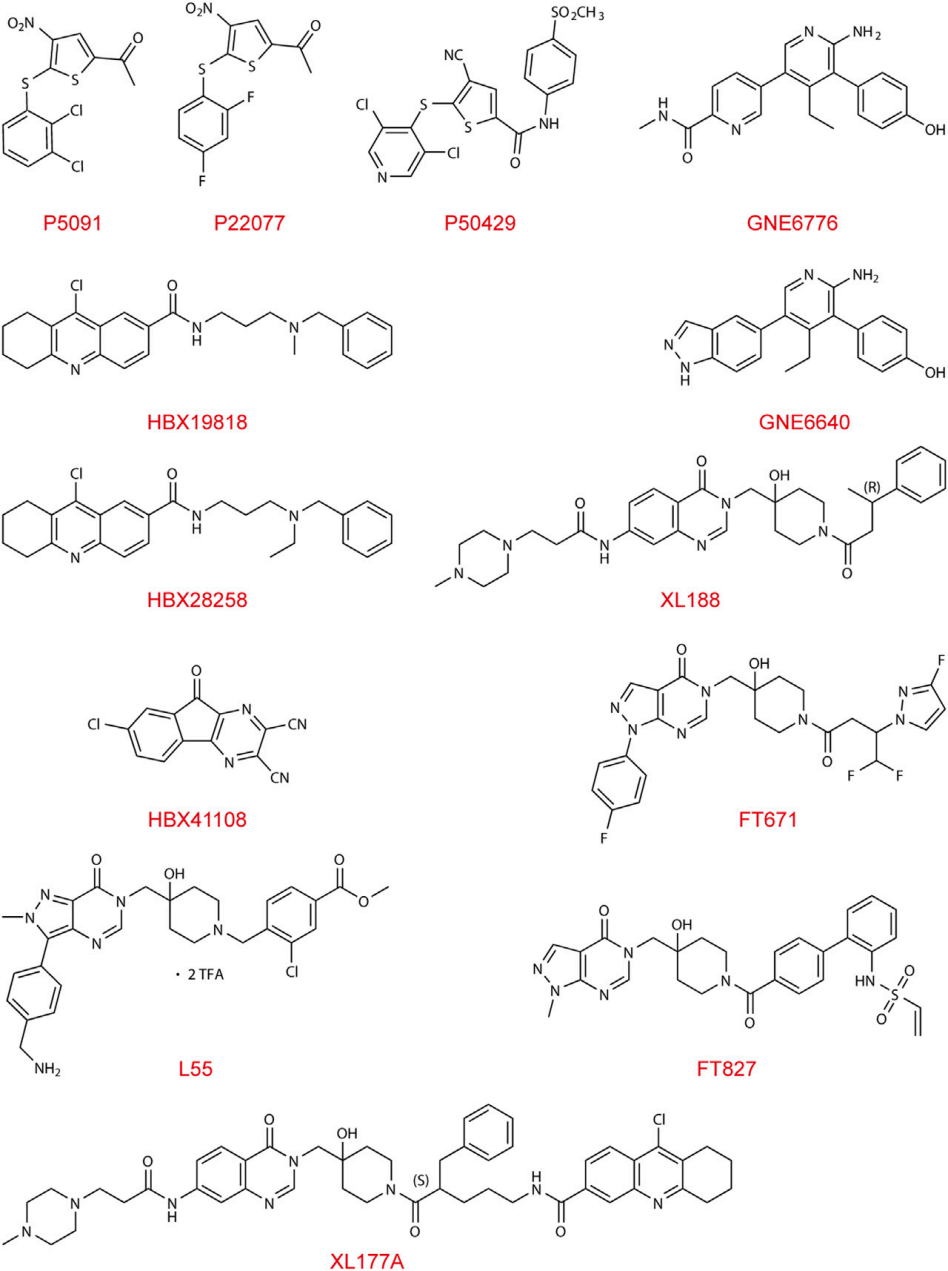
Chemical structures of USP7 inhibitors.

This section summarized the functions and characteristics of different types of USP7 inhibitors and provided their structures (Figure 2), hoping to inspire the design and development of USP7 small molecule inhibitors.

## Discussion

**FIGURE 3:**
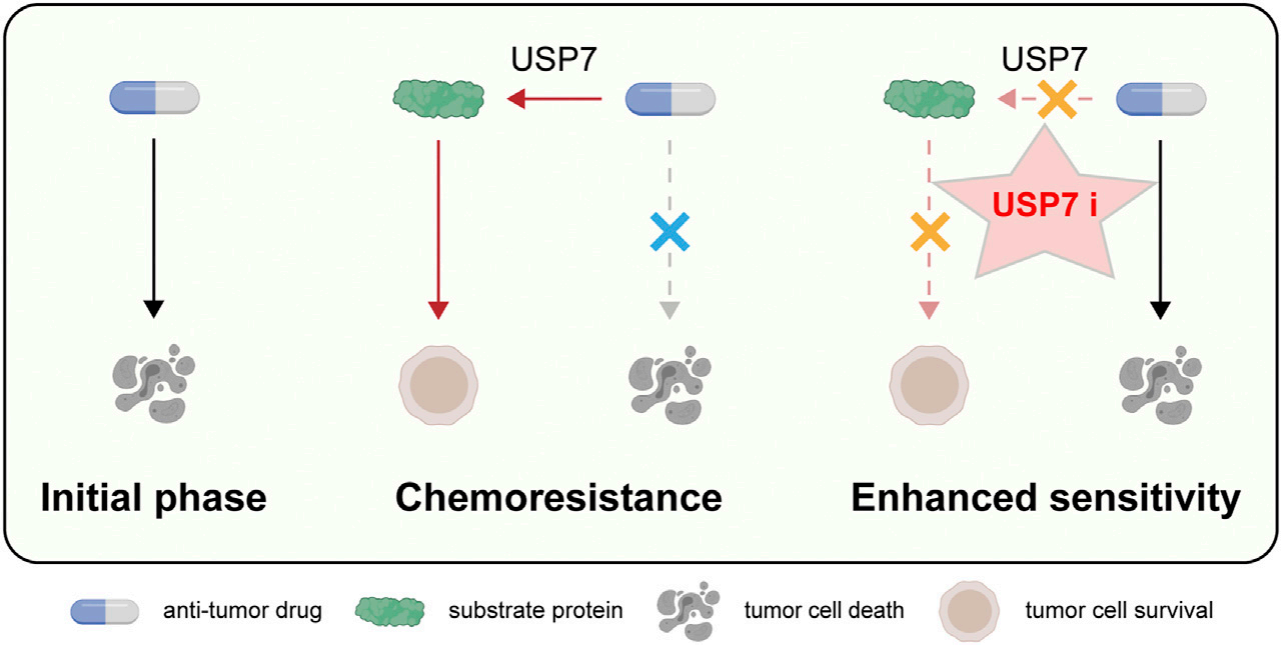
Schematic representation of targeting USP7 to overcome resistance to anti-tumor therapies.

In this review, we proceeded from the structure and function of USP7, further found that USP7 is closely related to tumor resistance, and proposed that targeting USP7 might be a new insight to overcome drug resistance (Figure 3).

In the past few years, extensive studies are carried in the structure, functions, and regulation of USP7. However, there remain questions to probe into. Firstly, despite its major function of USP7 to stabilize oncoproteins, there do exists some exceptions in which USP7 may have a role in cell cycle arrest and tumor suppression. For example, USP7 mediates the deubiquitination of the checkpoint protein CHFR. The case of another critical anti-tumor protein P53 is more complex. It’s widely believed that USP7 impairs p53 level via MDM2, but meanwhile, USP7 can remove ubiquitin off P53 in a direct manner. Therefore, it merits attention and study of the dual biological function of USP7. Secondly, further studies are needed to investigate how USP7 recognizes its diverse substrates. After the identifying the allosteric inhibitors including FT671 and GNE-6640, inhibiting USP7 activity by attenuating ubiquitin binding suggests a new strategy more applicable for engineering deubiquitinase inhibitors. Learning the mechanism of specificity allows us to manipulate the affinity of USP7 to a particular substrate pharmaceutically. For instance, it has been shown that the Phe409 sub-site of USP7 owns a great adaption to the ligands, and USP7 preferentially interacts with and cleaves ubiquitin moieties that have free Lys48 side chains. Thirdly, a large percentage of existing inhibitors take effect dependent on WT P53. With P53 mutation taking place in more than half of cancer cases, it’s necessary to study further whether and how USP7 works in a P53-independent manner.

Additionally, it will also be necessary to clarify whether the strategy of targeting USP7 to overcome cancer therapy resistance is beneficial for all cancer types or may have certain specificity. Based on the information summarized in Table 1 and other relevant references, we found a general rule that targeting USP7 can play a significant role in enhancing chemoradiotherapy sensitivity in various types of tumors, including hematological malignancies such as multiple myeloma, acute myeloid leukemia, and chronic lymphocytic leukemia, and solid malignancies such as breast carcinoma, lung cancer, colorectal carcinoma, prostate cancer, cervical cancer, and neuroblastoma. These tumors may prefer different genders, organs, and various growth stages, respectively; they also exhibit different degrees of malignancy. This theory’s generality is also manifested in the fact that targeting USP7 can help enhance tumor response to multiple therapeutic strategies, including traditional chemotherapy, molecular targeted therapy, immunotherapy, and radiation therapy. Hence, we believe that targeting USP7 will achieve universal benefits in the clinic for different tumors and different therapeutic strategies.

In summary, we have summarized the underlying mechanisms by which tumor cells develop resistance to anti-tumor therapies, provided theoretical support for targeting USP7 to overcome drug resistance, and further offered some inspiration for the design and development of USP7 inhibitors.
